# Accessible deep learning for automated segmentation of supported nanoparticles in electron microscopy

**DOI:** 10.1039/d6na00028b

**Published:** 2026-07-30

**Authors:** Christian Vedel Petersen, Marc Lindgaard, Nikolaj Nguyen, Peter Stanley Jørgensen, Yang Hu

**Affiliations:** a Department of Energy Conversion and Storage, Technical University of Denmark 2800 Kgs. Lyngby Denmark psjq@dtu.dk yanhu@dtu.dk

## Abstract

Fast and accurate quantification of the size and morphology of nanoparticles in electron microscopy images is essential for advancing heterogeneous catalysis and energy-conversion research, yet manual segmentation remains the main approach, which is time-consuming, subjective, and challenging to scale. Herein, we present an accessible and efficient deep learning model integrated into a fully functional analysis application for rapid segmentation and statistical quantification. Taken together, this work demonstrates that high-quality nanoparticle segmentation in electron microscopy images is feasible even with minimal annotated data and modest computational resources.

## Abstract 2

Herein, we present an accessible and efficient deep learning model integrated into a fully functional analysis application for rapid segmentation and statistical quantification.

## Graphical abstract

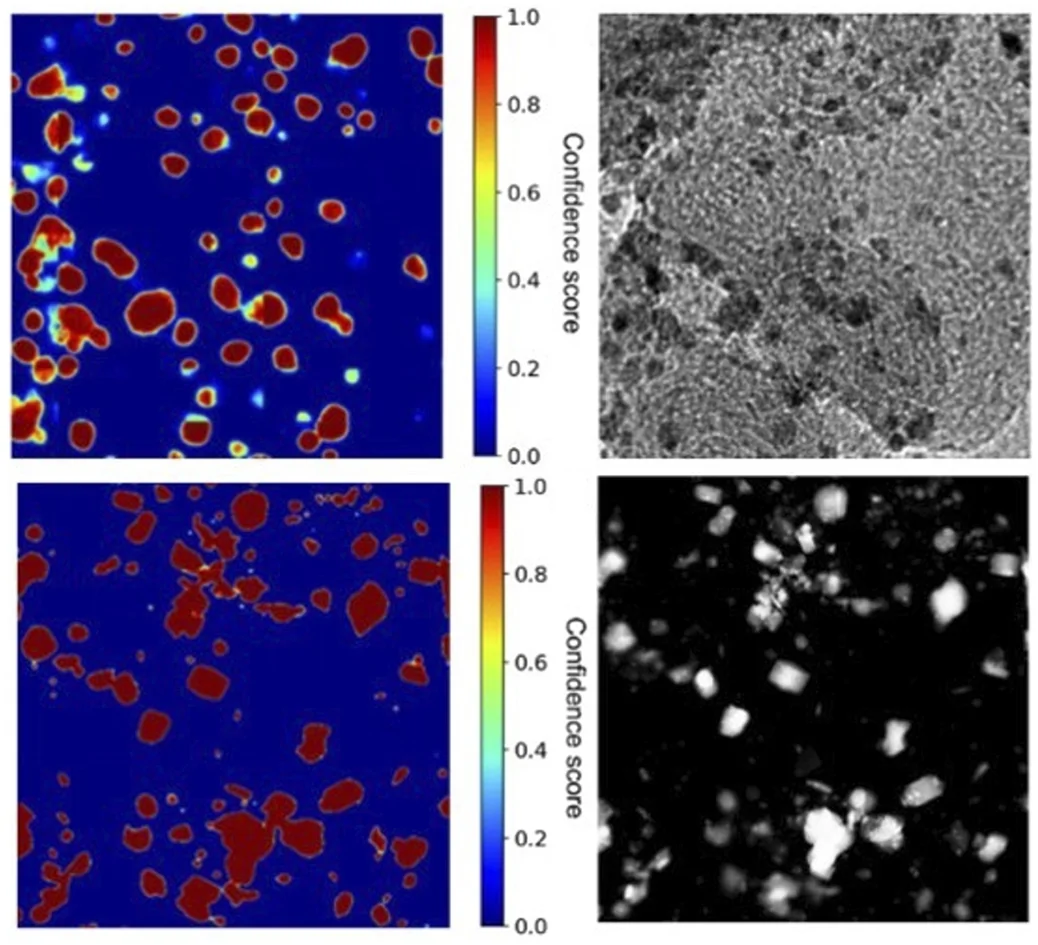

## Introduction

Supported nanoparticles, particularly those of metal and oxide, play a crucial role in heterogeneous catalysis, electrocatalysis, and energy conversion technologies.^1–4^ Their catalytic performance is closely tied to particle-level structural metrics such as size, dispersion, and morphology. Electron microscopy, including transmission electron microscopy (TEM), scanning TEM (STEM), and scanning electron microscopy (SEM), remains one of the most widely used techniques for resolving nanoparticle structure at high spatial resolution. However, extracting quantitative information from electron microscopy images traditionally requires extensive manual annotation of particle boundaries, typically executed through pixel-wise tracing or threshold-based segmentation. This process is time-consuming, subjective, and prone to substantial operator bias, especially when particle boundaries are ambiguous or when images contain noise, agglomerates, or overlapping structures. Variability in manual annotation introduces inconsistencies across studies, hampering reproducibility and slowing down materials development.

The emergence of deep learning and modern convolutional neural networks (CNNs) has significantly transformed the landscape of image segmentation, particularly in biomedical imaging, where U-Net architectures have become the predominant approach.^5–11^ Ronneberger *et al.* originally introduced U-Net as a compact, data-efficient model capable of producing pixel-level segmentations.^11^ U-Net architectures have also been explored in the context of nanoparticle segmentation; for example, Faraz *et al.* combined a U-Net model with object tracking to analyze dynamic morphological evolution during *in situ* TEM, demonstrating the applicability of this architecture to particle-level analysis.^12^ But the approach is tailored to dynamic *in situ* experiments and object tracking and therefore does not address general-purpose segmentation of static TEM images as considered here. Beyond U-Net, several general-purpose deep-learning models, such as Google's DeepLab and Meta's Segment Anything Model (SAM), offer strong performance on natural images and can, in principle, be adapted for nanoparticle segmentation.^13,14^ However, applying these architectures to low-contrast, low signal-to-noise data, like TEM, typically requires fine-tuning, custom preprocessing, or the construction of tailored inference pipelines, which requires significant software-specific expertise.^8,15–20^

Despite the growing interest in deep-learning-based nanoparticle analysis, few accessible applications exist that can reliably segment nanoparticles without additional programming or model development. While tools such as ANTEMA^8^ provide segmentation capabilities out of the box, their performance is typically optimized for a particular dataset and can decrease when encountering images outside that distribution. It is clear that there is a need for approaches that can be easily retrained or adapted to new nanoparticle systems.

In a typical materials research environment, researchers often possess hundreds of unlabeled electron microscopy images that frequently represent diverse materials with variable contrast and noise, posing a significant challenge: can reliable, precise nanoparticle segmentation be achieved with a compact model trained on minimal annotated data? This question is especially relevant for catalyst development, where rapid quantitative feedback on particle size distributions can guide synthesis optimization, degradation studies, and high-throughput materials screening. The present work addresses this challenge by developing a compact U-Net segmentation pipeline optimized explicitly for small, annotated datasets of target images. The resulting model is integrated into a desktop application enabling complete end-to-end analysis, including segmentation, annotation inspection, diameter and area computation, and batch processing. Additionally, the program supports training new models using custom datasets supplied by the user. The broader aim is to produce a deployable, user-friendly tool that accelerates nanoparticle characterization without depending on extensive training datasets or computationally intensive segmentation models.

## Results and discussion

In this work, we employed the U-Net deep learning architecture originally introduced by Ronneberger *et al.*^11^ The U-Net is implemented with four encoder blocks and four decoder blocks with batch normalization applied after each convolutional layer. The model input is a greyscale image of size 256 × 256. The output consists of two channels: one for the background and one for the foreground, each with a size of 256 × 256. To maintain the same resolution between input and output, zero-padding is applied to each convolution to avoid downsampling. Fig. S1 shows the model architecture. This deep learning model is implemented in the NanoAnalyzer application.

**Fig. 1:**
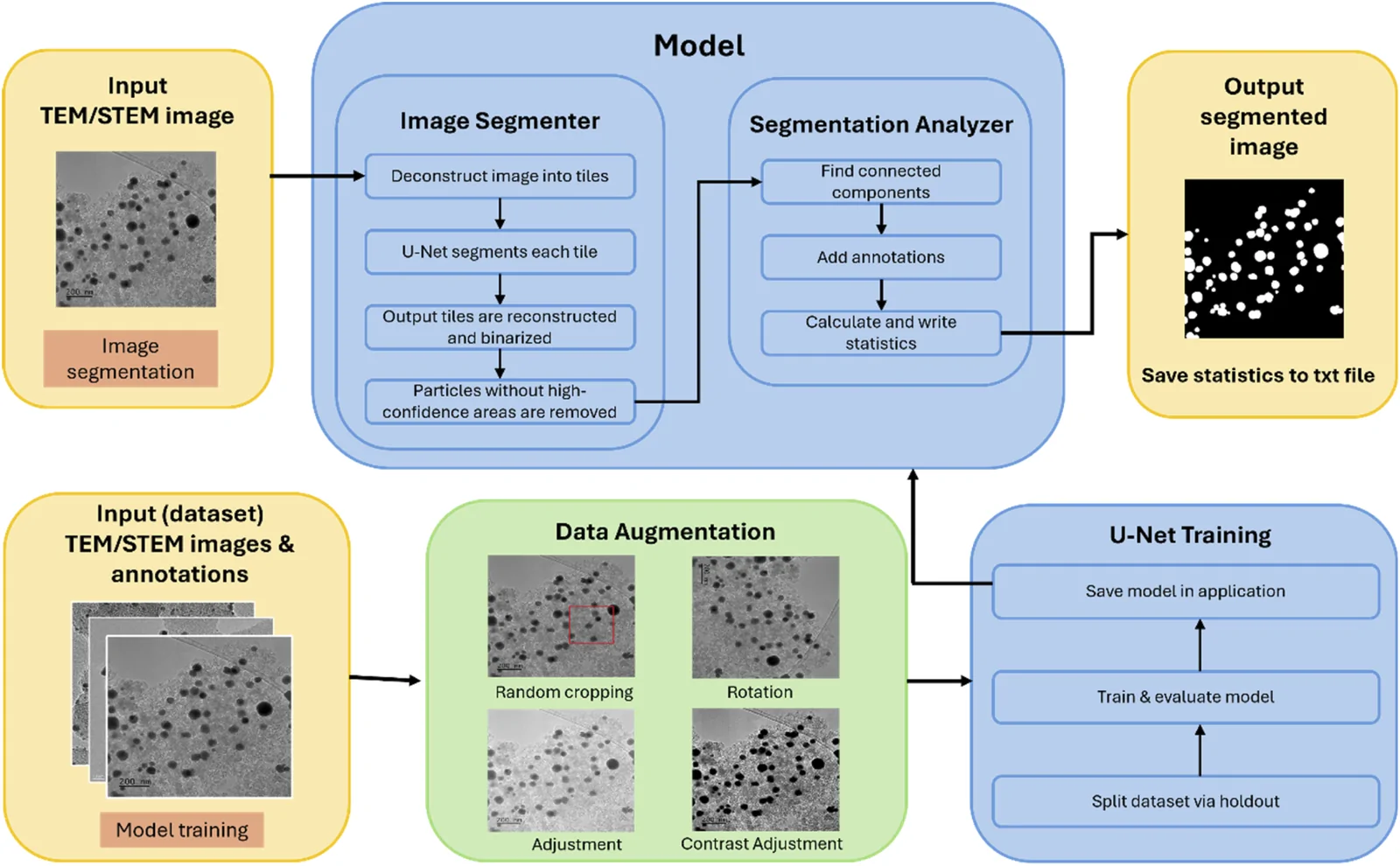
Overview of the NanoAnalyzer workflow. Colored boxes represent different application modules. Top: image segmentation from TEM/STEM images to a binarized mask and export of segmentation and statistics. Bottom: model training, where annotated images are augmented and split into patches; training/validation/test sets are generated and used to train and save a U-Net model.

A central aim of the application is to provide a seamless end-to-end segmentation workflow that delivers both robust masks and immediately usable quantitative outputs. An overview of the segmentation and training workflow is presented in Fig. 1, which illustrates several key application processes that impact overall segmentation quality and robustness. The overlapping, patch-based segmentation approach ensures smooth segmentation across the full image, without visible boundary artifacts. Low-confidence regions are filtered out during post-processing, thereby enhancing the overall segmentation quality, particularly in noisy images, where they significantly reduce false positives found in the smaller particle size ranges. The workflow also integrates an automated statistical step that labels each detected particle and estimates its size using the equivalent circular diameter (ECD) measurement. The resulting segmentation is accompanied by particle-level statistics, and an annotated segmentation output is provided to facilitate visual inspection and, if needed, user correction. Additionally, the model enables batch processing of images, thereby strengthening its practical applications by allowing for fast and consistent quantification across large datasets without requiring additional user intervention.

The lower portion of Fig. 1 illustrates the model training workflow, which directly affects the performance observed in the segmentation results. The inclusion of on-the-fly data augmentation exposes the model to a broad range of conditions similar to those in typical TEM and STEM applications, aiming to reduce the number of annotated images required to achieve a representative image environment. As a result, the trained model exhibits strong robustness to variations in illumination and background texture, consistent with the stable segmentation behavior shown for both high-contrast and low-contrast images – artificial augmentation shows virtually no difference in the resulting accuracy (see Fig. S2). The automatic split of images into training, validation, and test sets also ensures that the performance metrics displayed to the user reflect genuinely unseen data, supporting the reliability of the final model selection.

This training workflow is integrated into the NanoAnalyzer application, enabling users to train new models simply by providing a dataset of images and their corresponding annotations. Users can therefore generate models tailored to their specific imaging conditions. Although the model presented in this work was trained using TEM images of supported metal nanoparticles, the tool is sufficiently flexible to support model training on a wide range of datasets and applications. As the process requires no technical expertise, users can readily adapt the segmentation model to their own data, thereby improving performance and providing more reliable quantification of their particular samples. Using this training flow, a model was trained on a dataset comprising 20 bright-field TEM images and 5 STEM images, with data augmentation enabled. A screenshot of the NanoAnalyzer interface is provided in Fig. S3.

**Fig. 2:**
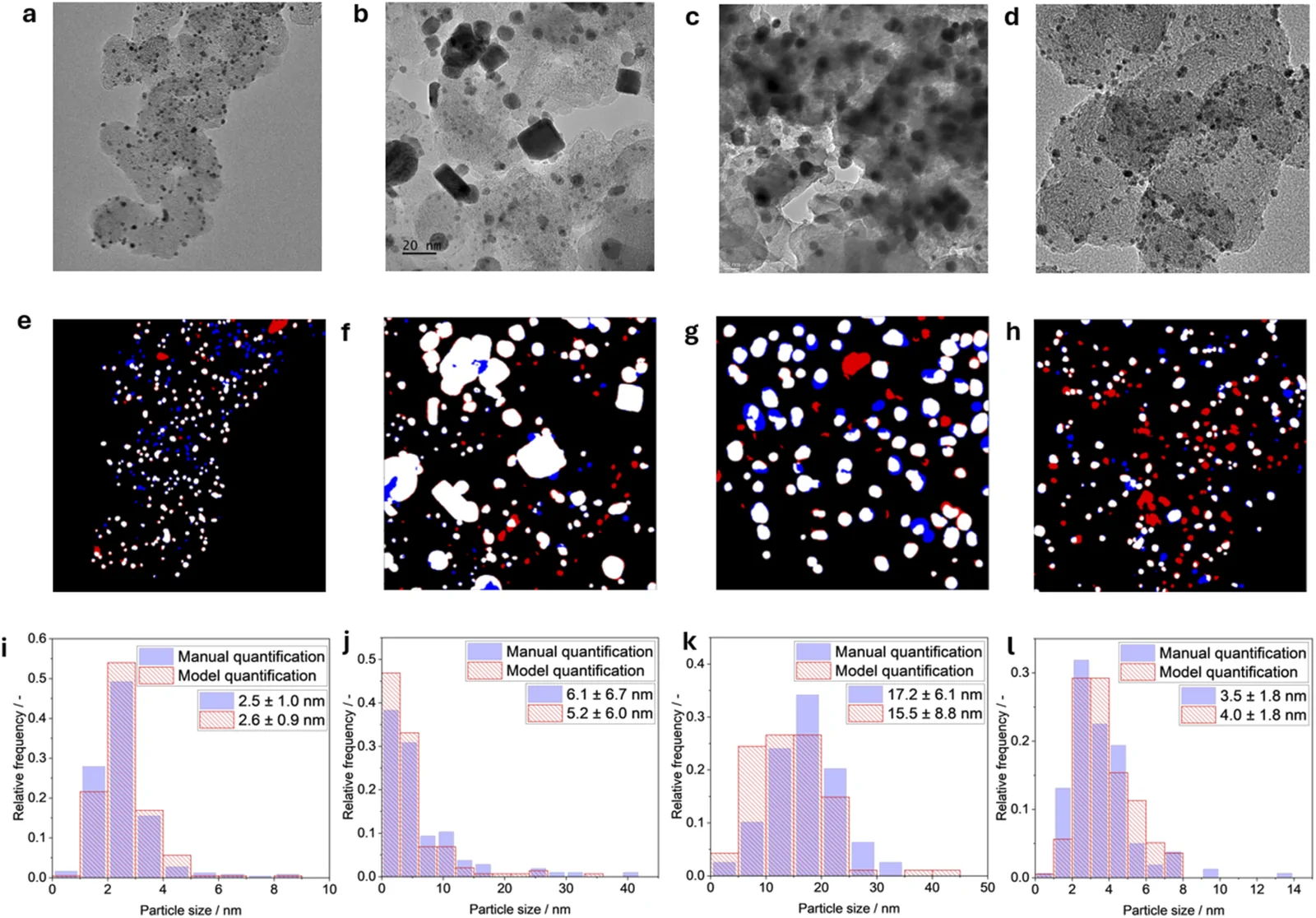
A collection of selected bright-field TEM images from various carbon-supported Pt or Pt-alloy samples, along with assorted graphs of analysis below the corresponding images. (a–d) Selected TEM images (e–h) “Comparison maps”, which overlay the model's segmentation output and the ground truth; the results of which are subsequently color-coded. (white = true positive, red = false positive, blue = false negative). (i–l) Particle size distribution histograms for each image. In each figure, the corresponding mean particle size and particle size analysis standard deviations are also denoted.

The performance and reliability of our segmentation method are evaluated by comparing its outputs with human-generated particle size distributions (PSDs), produced through extensive manual effort and careful inspection. Fig. 2 shows bright-field TEM images of a series of supported Pt and Pt-alloy nanoparticles acquired under diverse imaging conditions, together with their corresponding segmentation overlays and particle size distributions. The samples include a commercial sample of carbon-supported Pt nanoparticles (Fig. 2a), a mesoporous carbon-supported Pt_5_Ce nanoparticles of varied shapes (Fig. 2b), Vulcan XC-72R supported Pt_5_Ce particles alongside a secondary Ce–N–C amorphous phase (Fig. 2c), and Vulcan XC-72R supported Pt_*x*_Cr nanoparticles (Fig. 2d), which are synthesized by our group using a previously reported method.^21–23^ Across all samples, the model-generated PSDs align closely with manual measurements, confirming that the segmentation captures both mean particle sizes and distribution shapes. In low-contrast images, such as Fig. 2c, the model detects additional small particles compared with manual annotation, slightly lowering the mean particle size. This behaviour reflects the model's ability to identify weakly contrasted nanoparticles that are difficult to trace manually. Although the model outputs only binary masks—without phase separation for particles of varying contrast—the agreement in PSDs remains strong. To quantitatively evaluate the consistency, relative standard deviations extracted from model outputs were compared with manual values (Fig. S4). The excellent agreement between the two underscores that the model accurately captures both central tendencies and dispersion in particle size, validating its use for statistical nanoparticle analysis.

**Fig. 3:**
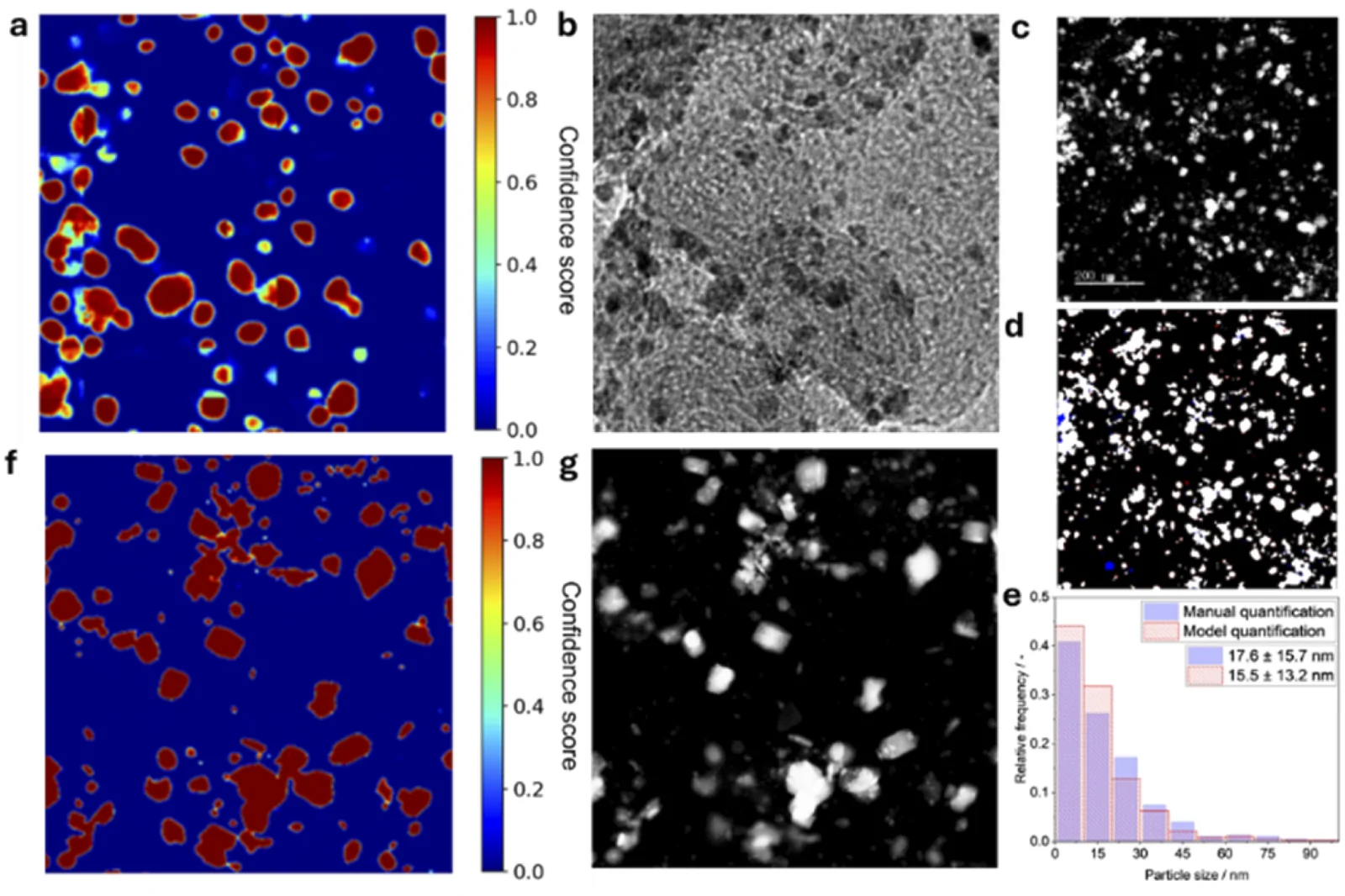
(a and b) Confidence map for a patch from sample 2d, along with the corresponding image patch. (c–e) A selected dark-field STEM images, along with its comparison map and PSD histogram. (f and g) Confidence map for a patch from sample 3b, along with the corresponding image patch. Confidence maps for the foreground class are computed by applying a softmax function to the model output.

The model also performs well under challenging imaging conditions that commonly complicate TEM analysis. Fig. 2 also features a variety of types of image disturbances that may be encountered during bright-field TEM image acquisition, leading to poor contrast between the foreground and the background (Fig. 2c), or inconsistent and ‘grainy’ background contrast (Fig. 2d). For the most part, the model seems to handle this quite well. Fig. 3a shows that areas of images with a foreground confidence score that is too low are discarded for analysis, resulting in a segmentation map that consists mostly of a high-confidence foreground environment. This results in a higher degree of spatial accuracy in the included particles, evident from the PSD, in exchange for a lower IoU. Even for images with a relatively poor IoU (as seen in Fig. 2k), the PSD remains close to that of the manual segmentation.

The model is highly versatile, capable of processing both TEM and STEM images without the need to train the model separately. Fig. 3c–e show a dark-field STEM image from a selected Pt_*x*_M/C sample and its corresponding segmentation analysis. Similar to the bright-field images, the segmentation agreement between the model's output and the human-produced data is relatively close, with PSDs that closely match. It is also evident that the higher contrast difference between the Pt_*x*_M nanoparticles and the carbon background supports more precise model segmentation. The comparison map in Fig. 3d shows very few instances of false-positive/negative regions, and Fig. 3f indicates that the foreground confidence scores assigned to the nanoparticle regions are almost exclusively around 1. This is also reflected in the high metrics of Fig. 3c, as shown in Table 1, compared to those of the bright-field TEM images.

**Table 1:** Intersection over Union (IoU) and other key metrics for each image showcased in the discussion, along with the mean metrics over the entirety of the dataset used for model training. Mean relative error is the mean absolute relative error in diameter estimation, reported as a fraction

Sample ID	IoU	Dice score	Precision	Recall	Mean relative error
2a	0.61	0.76	0.91	0.76	0.15
2b	0.88	0.94	0.79	0.91	0.086
2c	0.70	0.83	0.63	0.83	0.12
2d	0.52	0.68	0.67	0.73	0.13
3c	0.92	0.96	0.97	0.87	0.054
Mean (complete test set)	0.81	0.87	0.85	0.82	0.098

Importantly, sufficiently high segmentation performance can be achieved using only a limited fraction of the available annotated training data. To evaluate this, the dataset was first divided into training, validation, and test sets at the image level. Only after this split were the training images subdivided into four 512 × 512 patches, which were then randomly sampled at increasing fractions of the available training patches. This ensured that validation and test images remained independent of the training patches, while the experiment assessed how much annotated training material was required for the model to generalize to unseen images.

**Fig. 4:**
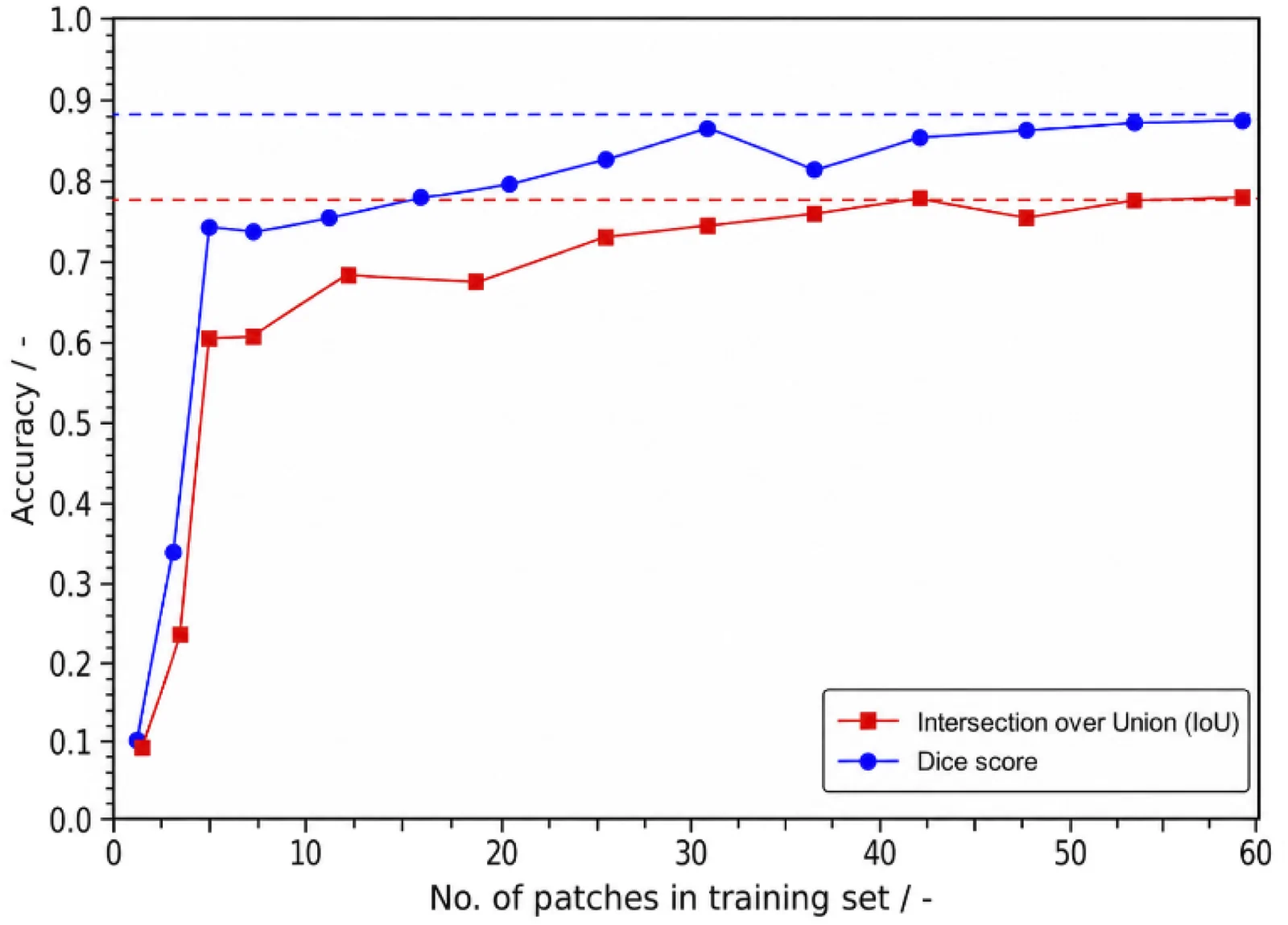
The number of patches included during training, as plotted against the resulting IoU and Dice score. The dotted lines represent the maximum accuracy that was achieved during all training runs.

Fig. 4 shows that the model accuracy increases rapidly when the first training patches are introduced, after which both IoU and Dice score approach a plateau. While increasing the number of training patches clearly improves performance in the early part of the experiment, the later plateau suggests that subsequently added patches contain partly redundant information under the present dataset conditions. Since the patches are sampled from image-level training data, even a limited subset can include examples from multiple images and expose the model to different particle appearances, contrast levels, and background textures. This highlights the importance of ensuring that annotated training data covers the relevant variation present in the target microscopy dataset.

The amount of training data required is therefore expected to depend on dataset heterogeneity. For datasets with consistent imaging conditions and particle morphology, relatively few representative patches may be sufficient. In contrast, datasets containing substantially different contrast profiles, support structures, particle sizes, or imaging modalities may require additional annotated examples before comparable performance can be expected.

It is also important to note that Fig. 4 reports IoU and Dice scores, which primarily measure pixel-level agreement. While these metrics are useful for quantifying spatial overlap, they do not fully describe the quality of the resulting particle analysis. Larger particles contribute more strongly to pixel-based scores because they occupy a larger image area, meaning that errors on small particles may be underrepresented. For this reason, pixel-level metrics should be interpreted together with particle-level quantities such as particle counts, mean particle size, size distribution, and relative size error, which provide a more application-relevant assessment of segmentation quality. Finally, segmentation and training times were benchmarked on two CPUs and two GPUs of varying performance levels. As summarized in Table S1, inference times on CPUs range from 7 to 20 s and 0.35 to 0.97 s on GPUs, indicating that CPU-only segmentation is practical. Training times range from 13 to 18 hours on the CPU and from 10.2 and 121 minutes on the GPU, so GPU usage is strongly recommended for model training. Due to the challenges in labelling overlapping particles, the presented segmentation model does not support individual segmentation of overlapping particles. However, this functionality could be added by extending the U-Net model with additional decoders^24^ and training it with multiclass labelled data where the overlap has been accurately annotated.

## Conclusion

In this work, we demonstrate that sufficiently accurate and fast segmentation of supported nanoparticles from electron microscopy images can be achieved using a compact deep-learning pipeline trained on a small number of annotated images. By combining patch-based U-Net segmentation with carefully designed augmentation strategies—such as random cropping and rotational transformations—the model attained strong quantitative performance despite complex imaging conditions. Qualitative analysis highlights the model's ability to segment particles in varied imaging conditions, while also revealing opportunities for improvement in handling ultra-small particles, low-contrast regions, and overlapping particles. The integration of the trained model into a user-friendly analysis application enables rapid segmentation, particle detection, and statistical reporting on both CPUs and GPUs, making the tool accessible to a broad range of materials researchers. By lowering the barrier to automated nanoparticle analysis, this work provides a practical foundation for accelerating catalyst development and electron microscopy workflows. Future improvements—including instance segmentation, uncertainty-aware learning, active annotation, and fine-tuning on diverse materials systems—could open even more exciting possibilities.

## Conflicts of interest

There are no conflicts to declare.

## Supplementary Material

NA-008-D6NA00028B-s001

## Data Availability

The code for ‘NanoAnalyzer’, along with the dataset and annotations can be found at https://github.com/ExtraUnity/NanoparticleAnalysis. The version of the code employed for this paper is version 1.1.3 (found under the ‘Releases’ tab). Supplementary information (SI) is available. See DOI: https://doi.org/10.1039/d6na00028b.
