# Supplementary material for: Accessible deep learning for automated segmentation of supported nanoparticles in electron microscopy
Source: Nanoscale Adv. 2026 Jul 30;8(17):4770–5. doi: 10.1039/d6na00028b (PMC13440630; doi:10.1039/d6na00028b)
Supplement: Supplementary file 1 [file NA-008-D6NA00028B-s001.pdf]

Supplementary Information (SI) for Nanoscale Advances.
This journal is © The Royal Society of Chemistry 2026

Supporting Information for "Accessible Deep Learning for Automated Segmentation of Supported Nanoparticles in Electron Microscopy"

Christian Vedel Petersen,[a†] *Marc Lindgaard,*[a†] *Nikolaj Nguyen,*[a] *Peter Stanley Jørgensen,*[*a] *and Yang Hu*[*a]

[a] *Department of Energy Conversion and Storage, Technical University of Denmark, 2800 Kgs. Lyngby, Denmark*

† Both authors contribute equally to this work.

Sample syntheses. The samples used in this study are a series of carbon-supported Pt- or Pt-alloy nanoparticles. Apart from the commercial sample, all the other samples were synthesized using a slight variation of the solid-state synthesis procedure previously reported by our group, [1-3] in which size-controlled Pt-alloy nanoparticles are synthesized using the use of nitrogen-containing organic compounds to facilitate the in-situ formation of a polymeric, carbon-supported C-N network, which coordinates the metal ions in the precursor salts, thus protecting them from uncapped oxidation and allowing for control of the reduction conditions. [2, 3]

Transmission electron microscopy imaging and manual particle segmentation. Transmission electron microscopy (TEM) is performed using the FEI Tecnai G2 20 S-TEM, equipped with a LaB6 emitter and operating at an accelerating voltage of 200 kV. Images are acquired using a bottom-mounted TVIPS TemCam-XF416 CMOS camera at a resolution of 4 K × 4 K. All the microscope elements are controlled digitally via the internal Tecnai user interface. High-angle annular dark-field scanning transmission electron microscopy (STEM) images were recorded at 300 kV using an FEI Titan Analytical 80-300ST TEM equipped with a spherical aberration corrector and an X-Max TLE energy-dispersive X-ray spectroscopy detector. The manual quantification of particle diameter and particle size distribution (PSD) is performed using the ImageJ image manipulation software. Each particle is measured individually, assuming spherical particle shape.

Deep learning architecture. To support the segmentation of images larger than 256×256 pixels, a tiling strategy is employed. At inference, the input image is divided into overlapping patches of 256×256 pixels, using a stride of 200 pixels, to ensure overlap between adjacent patches. Each patch is then segmented by the model. Afterwards, the segmented patches are stitched back together, and overlapping regions are averaged to ensure seamless transitions and minimize

artifacts at patch borders. To allow segmentation of arbitrarily sized images, mirror padding is used. The model is trained using the Adam optimizer with a cross-entropy loss function. For GPU training, the batch size is set to 32, while for CPU training, it is set to 8 to limit memory usage. ReLU is used as the activation function. By default, it uses a learning rate of 10^{-4} and trains for a maximum of 150 epochs with a patience of 25, although these parameters are customizable in the application interface.

Data augmentation: Due to the limited dataset size, the application relies heavily on data augmentation to enhance the model's robustness. Each image in the dataset is first downscaled, if necessary, to a maximum of 1024 x 1024 pixels. The dataset is then split into training, validation, and test sets at the image level. For the data size experiment, only the images assigned to the training set are subsequently subdivided into four non-overlapping 512×512 patches. The different training-set sizes reported in Figure 4 are obtained by randomly sampling increasing fractions of these training patches. This ensures that validation and test images remain independent of the training patches, while the experiment evaluates how much annotated training data is needed to achieve robust segmentation. A visualization of this tiling strategy is shown in Figure S5a. During training, the U-Net operates on 256×256 inputs. For each 512×512 patch, a random 256×256 crop is generated on the fly, introducing translational augmentation while maintaining a consistent input size, which has been found to yield the best accuracy.[4] This random cropping is illustrated in Figure S5b. Additional augmentations are applied to these crops, namely random 90° rotations, and random brightness and contrast adjustments. Random rotation is used as it is generally one of the most efficient data augmentation methods for deep learning when image objects do not have a predetermined orientation, such as nanoparticles.[5] Random brightness and contrast adjustments account for the fact that microscopy images are often preprocessed manually to enhance particle

visibility, a subjective process that can introduce significant variability. Data augmentation is applied "on the fly" during data retrieval, meaning only the original images are stored, and augmentations are applied dynamically at each access. Over many epochs, this effectively mimics having a large set of precomputed augmented images, but with significantly reduced storage requirements and greater diversity than would be obtained from a fixed set of precomputed crops.

Model training. NanoAnalyzer supports training new models. This allows the user to create specialized models tailored to their own custom TEM or STEM datasets, making segmentation and analysis more accurate for their own data. The goal of this is to make training a deep learning model accessible for non-technical users. Therefore, all hyperparameters are set at the default values. The user simply needs to supply the dataset. A dataset is composed of two folders: one containing images, and another containing their respective masks (annotated images). The dataset will be split by default into a training set, a validation set, and a test set, using a split of 60/20/20. Optionally, a separate test set can be input into the program, in which case the training and validation split will be 80/20. When a dataset is supplied, the program first checks if the dataset is valid. This involves first validating that the images and masks have the exact image dimensions. The program also verifies that the masks supplied are binary images (and corrects near-binary pixels that might have been created during downscaling). In case high-resolution images are supplied, the program automatically downscales the image and mask to a maximum of 1024x1024. This is to be consistent with segmentation at inference time, where images are also downscaled to 1024x1024 for computational efficiency. It is important to note that the quality of the annotation has a significant impact on the performance of the trained model. While not every particle needs to be annotated precisely, the model's accuracy might suffer if annotations are highly incomplete.

Pretrained model. The pretrained model was trained using a dataset of 20 bright-field TEM images and 5 STEM images, taken from selected $P_{tx}M/C$ samples. The images were annotated manually using the image processing software FIJI, assisted by the LabKit plugin.[6] The model is provided to give a baseline for segmentation and analysis of images and to allow immediate usability of the program. The model is trained on a range of different TEM/STEM images to enhance its accuracy across multiple types of samples; however, the model's accuracy depends on the similarity between the training images and those used for inference.

Pre- and postprocessing. Before images are fed to the U-Net model, light preprocessing of the images is done. Z-score standardization is applied before training to place pixel intensities on a common scale, which is widely recommended to stabilize gradients and increase convergence speed in many machine-learning models. At inference time, the input image is downscaled to a maximum of 1024 x 1024 pixels for computational efficiency and then segmented using the described tiling strategy. For arbitrarily sized images, their dimensions might not be evenly split by these overlapping patches. To circumvent this, images are padded to the correct size using mirror-padding. After the full segmentation output is collected, it is binarized in the following way. The per-pixel foreground and background class probabilities are obtained by applying a softmax to the U-Net output logits. A candidate binary segmentation map is then computed by the argmax operation between the background and foreground classes of the output. Each connected component in the candidate map is treated as a particle candidate. Additionally, "seed" pixels with a foreground probability above 0.85 are identified. The candidate particles are filtered and only accepted if they contain such a seed. Otherwise, it is rejected and removed from the segmentation map. This region-level confidence filtering is employed instead of a simple argmax, as it significantly reduces the number of small, noisy fragments and yields a cleaner segmentation

output, leading to more reliable particle statistics. For statistical analysis, the program identifies each particle using connected component analysis. It then extrapolates the area for each particle, determined by total pixel area, and estimates the diameter, determined by the Equivalent Circular Diameter (ECD) measurement. This data is written to a text file (.txt) along with a summary table.

Supporting figures and tables.

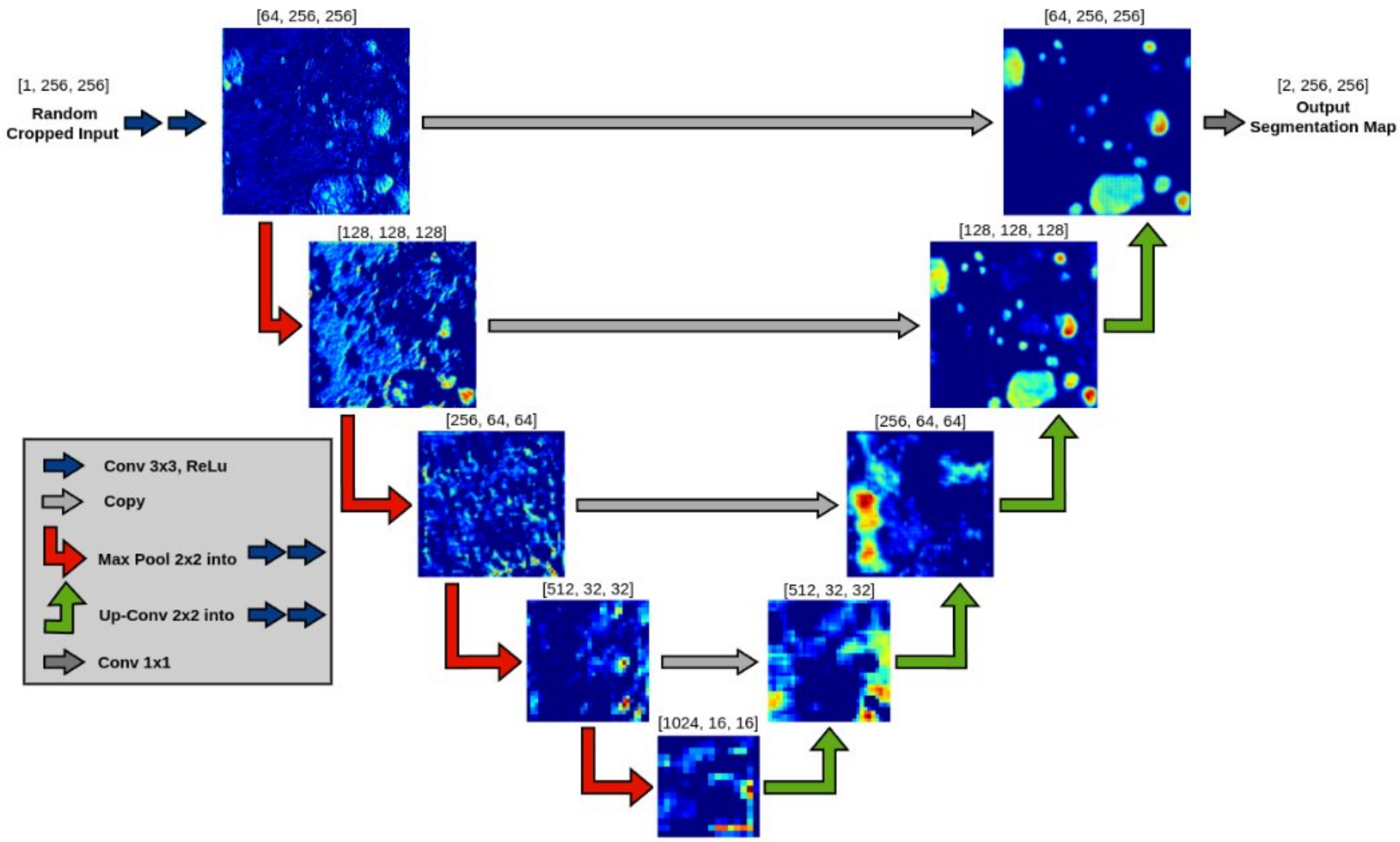

Figure S1. U-Net architecture used in this work. Feature map shapes [channels, height, width] are shown above each layer; arrow colors correspond to the operations in the legend.

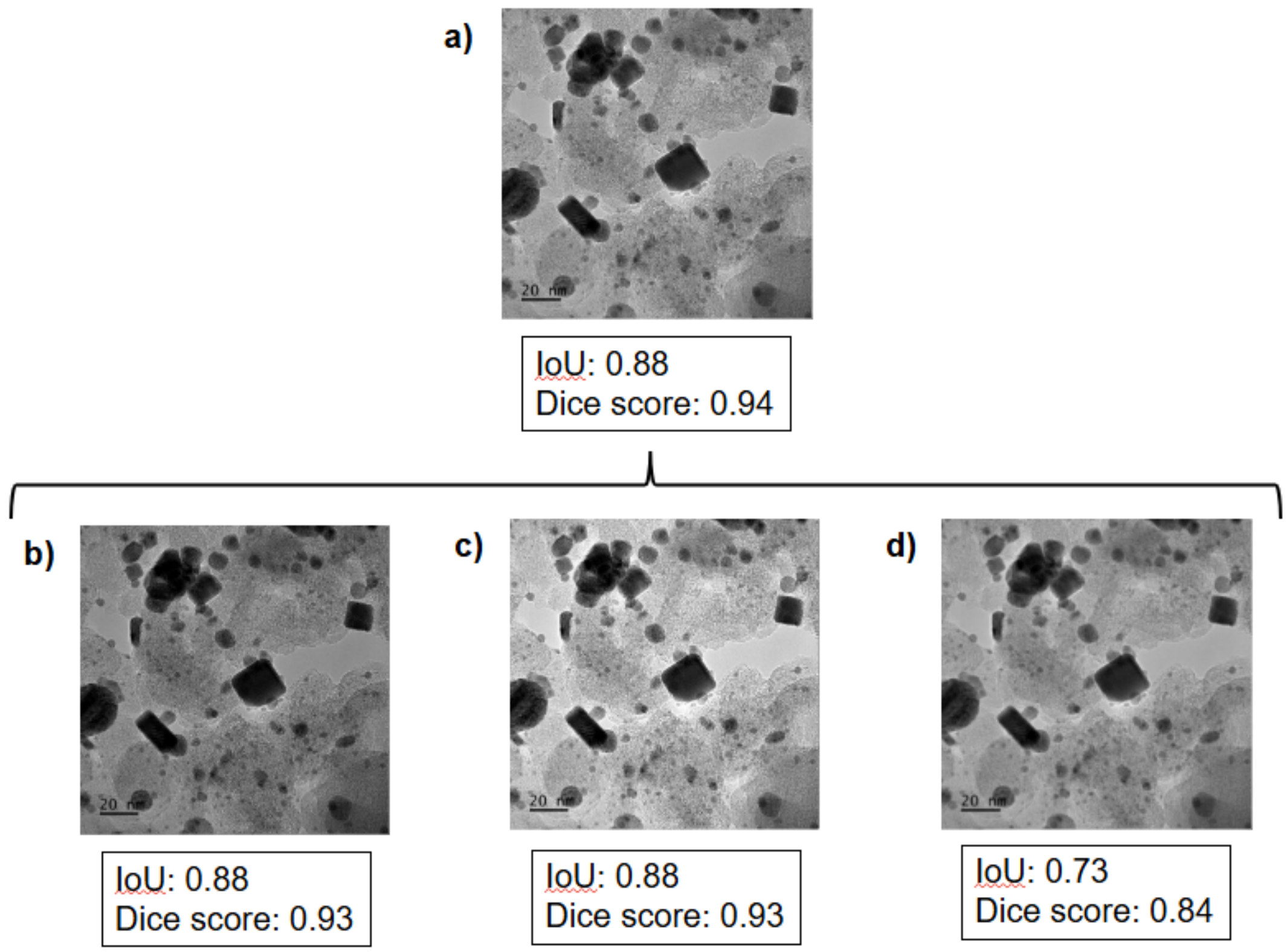

Figure S2. The segmentation results of applying a series of image manipulations to the TEM image presented in Figure 2b. (a) original image, (b) brightness and contrast decreased by 20%, (c) brightness and contrast increased by 20%, (d) Gaussian blur applied.

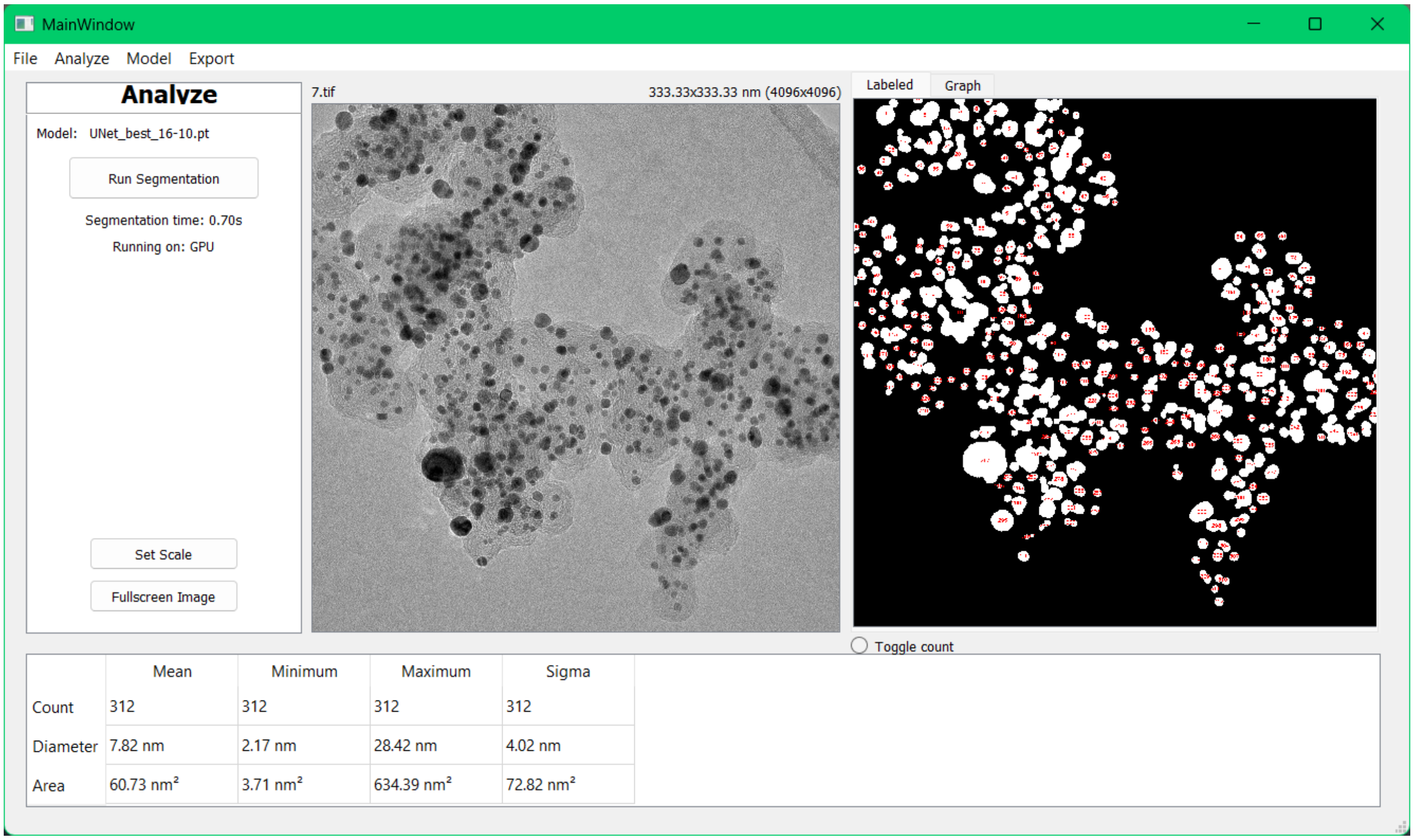

Figure S3. Image of the UI interface after having run a segmentation in GPU mode, running on Windows 11.

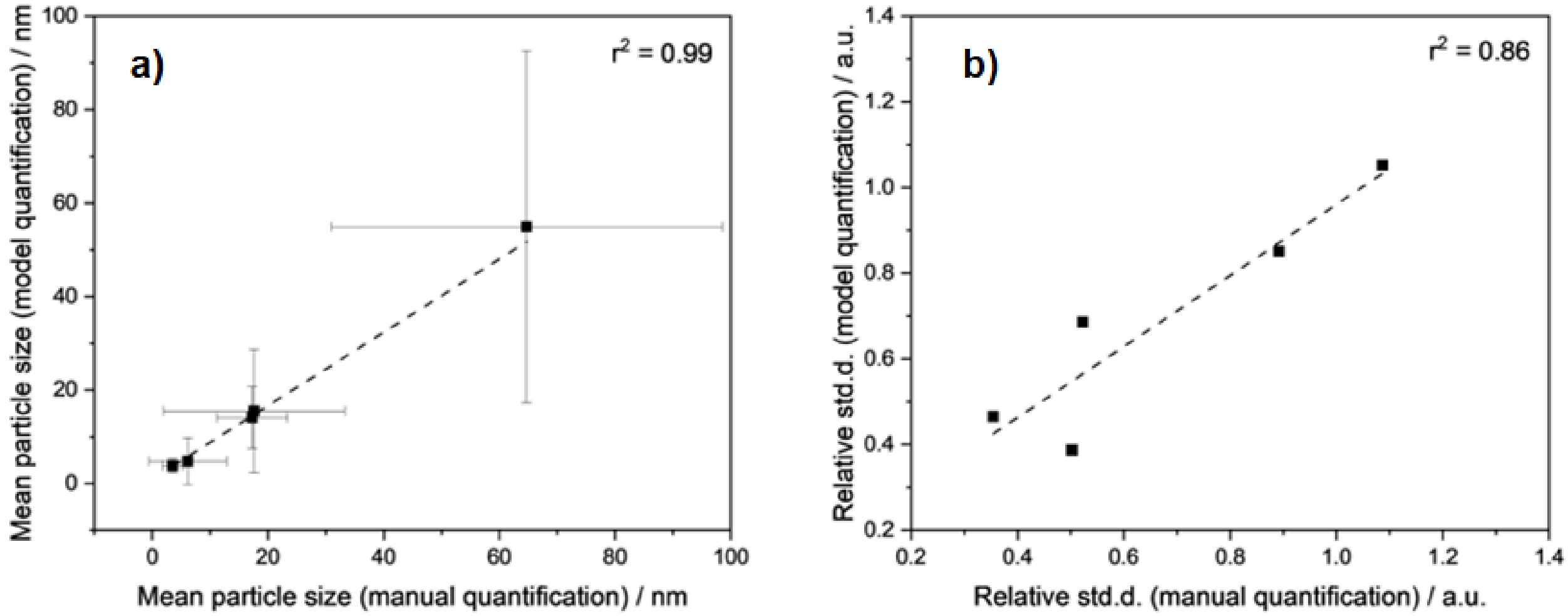

Figure S4. (a) The mean particle size, as obtained from the ground truth, against the mean particle size, as obtained from the model segmentation output. (b) The relative standard deviation (σ/μ), as obtained from the ground truth, against the relative standard deviation, as obtained from the model segmentation output.

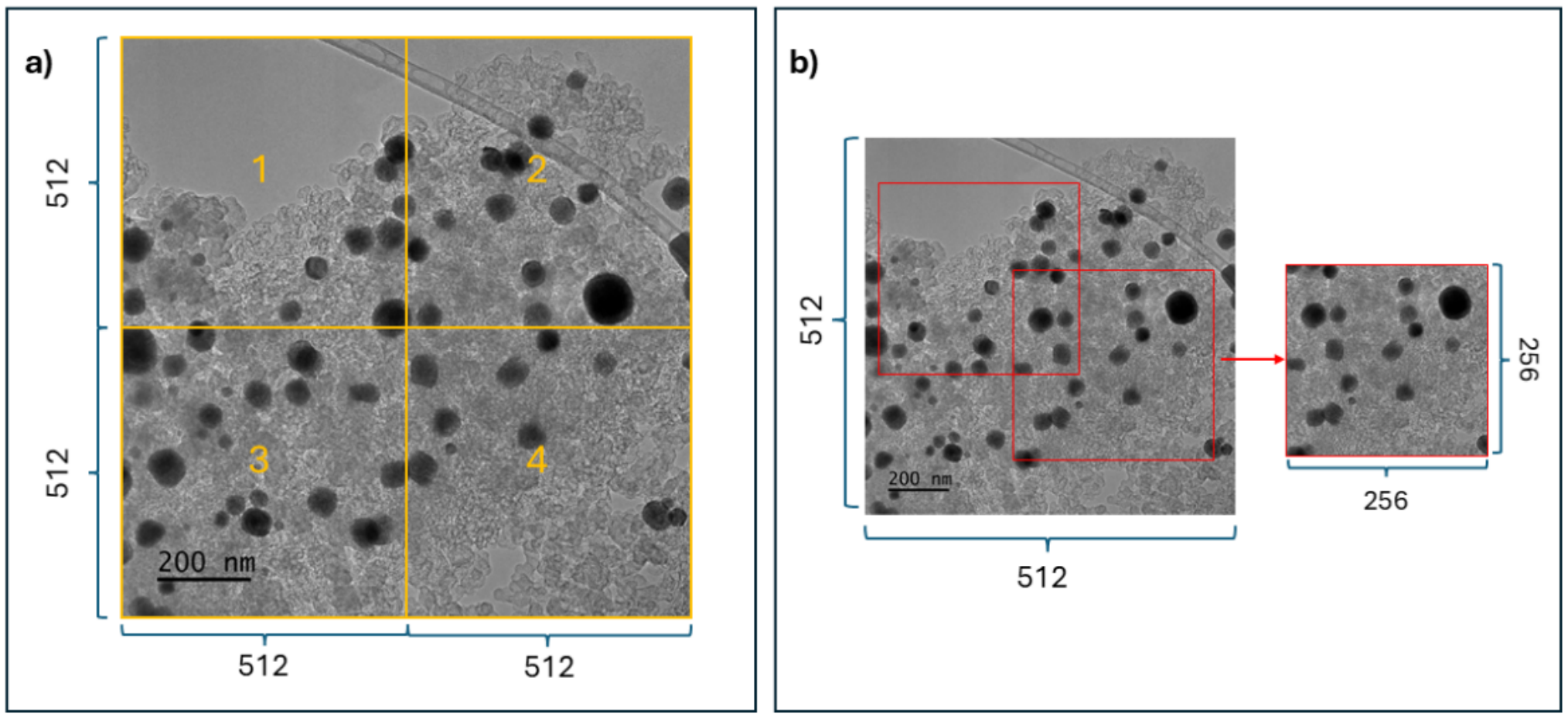

Figure S5. Tiling and augmentation strategy. (a) For the data size experiment, the training set only is subdivided into four non-overlapping 512x512 patches, from which the training data is sampled from in the experiment scenarios. (b) During training, the U-Net operates on 256×256 inputs obtained as random crops from these 512×512 patches, combined with on-the-fly rotation and brightness/contrast augmentation.

Table S1. Benchmarking results from the segmentation and training features of the NanoAnalyzer application. Segmentation time is calculated as the mean of 10 different image segmentations. Training time is taken from a sample training run done on each of the processors.

Mode	Processor	Mean image segmentation time / s	Model training time / min
CPU	AMD Ryzen 7 PRO 6850U @ 2.7GHz	6.91	810.6
CPU	Intel Core i5-1135G7 @ 2.4GHz	20.4	1080.0
GPU	NVIDIA RTX 2060	0.97	121.0
GPU	NVIDIA A100-PCIE	0.35	10.2

Size-stratified results

To evaluate whether the model performance depends on particle size, the segmentation results were stratified into small, medium, and large particles based on the equivalent circular diameter (ECD). The 33rd and 66th percentiles of the ECD distribution were used as bin boundaries, dividing the particles into three approximately equally sized groups. The size-stratified metrics are shown in Table S2.

The results show that segmentation performance generally improves with increasing particle size. Large particles achieve the highest overall agreement, while small particles show lower IoU, Dice score, precision, and recall. This trend is expected, as larger particles occupy more pixels and often have more clearly defined boundaries, whereas small particles are more affected by low contrast, boundary uncertainty, and small annotation differences. The mean relative error follows the same general trend, indicating that size estimation is most challenging for the smallest particles. These

results support the overall applicability of the model across the particle size range, while showing that small particles remain the most difficult to segment and quantify accurately.

Table S2. Size-stratified segmentation performance. Particles were divided into small, medium, and large bins based on the 33rd and 66th percentiles of the equivalent circular diameter (ECD) distribution. Mean relative error is reported as the mean absolute relative error in ECD.

Size bin	IoU	Dice score	Precision	Recall	Mean Relative Error
Small	0.71	0.79	0.73	0.70	0.11
Medium	0.73	0.91	0.87	0.87	0.10
Large	0.83	0.98	0.95	0.92	0.086
Overall	0.81	0.87	0.85	0.82	0.098

Cross-annotator comparison

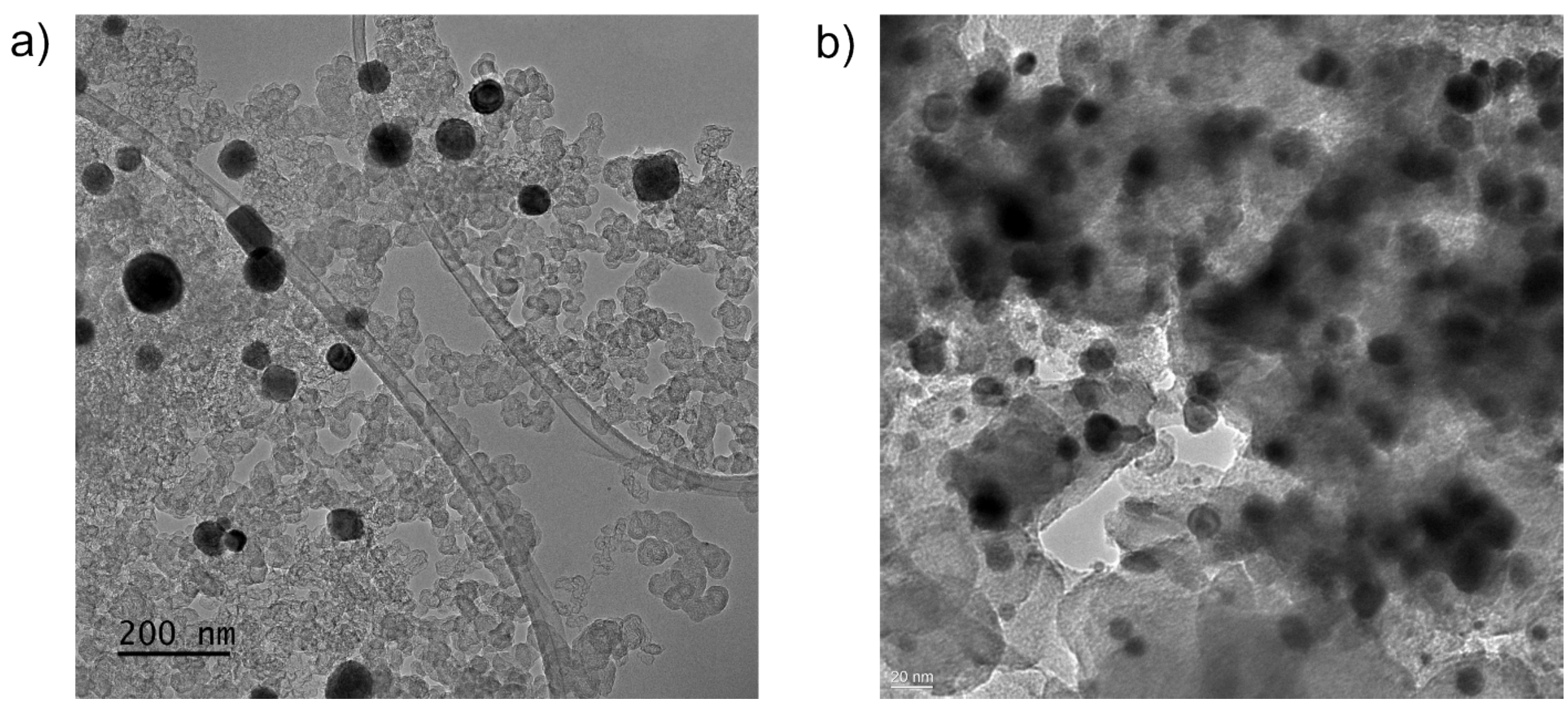

Figure S6. Images used for cross-annotator comparison. Two representative images were independently annotated by two annotators to estimate manual annotation agreement. The corresponding agreement metrics are reported using IoU and Dice score.

To assess the consistency of the manual annotations in the dataset, two representative images were chosen for a cross-annotator comparison. Each image was independently annotated by two

annotators, and the resulting binary masks were compared using IoU and Dice scores, as shown in Table S3. For the image shown in Figure S6a, a high agreement is seen, with an IoU of 0.93 and a Dice Score of 0.96. For the more challenging image shown in S6b, an IoU of 0.69 and a Dice Score of 0.82 was measured. This indicates that the uncertainty in an annotation image is highly dependent on the image and becomes more pronounced for difficult images with lower contrast, overlapping structures, and less-defined particle boundaries. These results provide a useful perspective in interpreting the model performance, as part of the observed disagreement between the model prediction and the ground truth may reflect the inherent ambiguity in the annotation process.

Table S3. Results from the cross-annotator comparison. Agreement is measured in IoU and Dice Score for the best direct interpretation in relation to model performance.

Image	IoU	Dice Score
**S6a**	**0.93**	**0.96**
**S6b**	**0.69**	**0.82**

Comparison with classical thresholding methods

To evaluate whether nanoparticle segmentation could be achieved using classical thresholding methods, we implemented two conventional algorithms: Otsu thresholding and adaptive thresholding. We then assessed their accuracy on the full volume of TEM and STEM images respectively. The results are seen in table S4

For the TEM images, both thresholding methods showed limited segmentation performance. Otsu thresholding achieved the best IoU and Dice score of these two. Although models showed moderate recall performance, the object-level precision was very low, indicating that the

thresholding methods produced a lot of false positives. This suggests that simple intensity-based segmentation is poorly suited for TEM images with variable background contrast, support structures, and weakly defined particle boundaries.

For the STEM images, Otsu thresholding performed substantially better than adaptive thresholding, achieving an IoU of 0.61 and a Dice score of 0.75. This indicates that conventional thresholding can be partially effective for high-contrast STEM images where the particles are clearly separated from the support. However, object-level precision and recall remained limited and the mean relative particle-size error was higher than for adaptive thresholding. These results show that thresholding performance is highly image-dependent and sensitive to imaging modality and contrast conditions.

Overall, the baseline comparison supports the need for more sophisticated learning-based models. While simple thresholding may produce acceptable results for selected high-contrast STEM images, precision and recall metrics both lacked, and it does not provide robust segmentation across both TEM and STEM data. The U-Net based approach reported in the main manuscript provides a more robust and flexible segmentation approach across the dataset.

Table S4. TEM Results

Method	IoU	Dice score	Precision	Recall	Mean Relative Error
Adaptive thresholding	0.23	0.36	0.027	0.61	0.16
Otsu thresholding	0.39	0.54	0.043	0.55	0.17
**STEM Results**					
**Method**	**IoU**	**Dice score**	**Precision**	**Recall**	**Mean Relative Error**
Adaptive thresholding	**0.19**	**0.32**	**0.54**	**0.63**	**0.15**
Otsu thresholding	**0.61**	**0.75**	**0.56**	**0.45**	**0.25**

References

1. Q. Zhou, J. O. Jensen, L. N. Cleemann, Q.-F. Li and Y. Hu, *Advanced Sensor and Energy Materials*, 2022, **1**, 100025.
2. Y. Hu, J. O. Jensen, P. Norby, L. N. Cleemann, F. Yang and Q. Li, *Chemistry of Materials*, 2021, **33**, 535–546.
3. Y. Hu, J. O. Jensen, L. N. Cleemann, B. A. Brandes and Q. Li, *Journal of the American Chemical Society*, 2020, **142**, 953–961.
4. S. P. Kannojia and G. Jaiswal, *Int. J. Comput. Sci. Eng*, 2018, **6**, 451–456.
5. R. Hao, K. Namdar, L. Liu, M. A. Haider and F. Khalvati, *J Digit Imaging*, 2021, **34**, 862–876.
6. M. Arzt, J. Deschamps, C. Schmied, T. Pietzsch, D. Schmidt, P. Tomancak, R. Haase and F. Jug, *Frontiers in computer science*, 2022, **4**, 777728.
